# Philadelphia Letter

**Published:** 1869-06-15

**Authors:** E. R. Hutchins

**Affiliations:** Philadelphia


### ORIGINAL COMMUNICATION.

PHILADELPHIA, *May* 27, 1869.

EDITOR CHICAGO MEDICAL JOURNAL:

Necessary absence, producing an unusual amount of work, forbade my writing you before. Material was ample, but opportunity *rare.* You know doctors, especially, are very apt to jump on a hobby and *ride* it too, sometimes, unfortunately, to death. My hobby was chosen sometime ago, and though I have at times ridden hard, and not unfrequently pranced my steed before your readers, still I am glad to say it is renewing strength daily. Mine is a speculum. With it how mnch good has been accomplished for suffering womankind. In this letter I have to relate three cases of procidentia and prolapse of the uterus, all of which were completely cured by the operation, *known* (only in books) under the term *episoraphia.* This operation for the permanent relief of patients affected as before-mentioned, was first proposed by M. Girardin, and resembles very closely Dupuyrens or Hey's operation for prolapsus ani. It has been performed now by very many in our own country, as well those abroad. The operation consists in removing a portion of the vaginal mucous membrane with scissors or bistoury, and then bringing the edges together with the quilled suture, thus making the extent of vaginal openiug as much less as the amount removed. A prolapsed uterus by no means is dependent on the laxity and elongation of the ligaments. While these ligaments *aid* in maintaining the uterus in proper position, the *walls of the vagina* perform this function especially.

Prolapse, either in greater or less degree, *must* result from an uterus largely hypertrophied, and of course increased in weight as well as in size. And in very many cases, as soon as the inflammation and hypertrophy are cured, the prolapse also is cured. But if this does *not* remedy the evil, we must have recourse to this operation. Within the past few months three cases have come under my observation and treatment. The first is that of Mrs. B., age 37, mother of three children. Has been a resident of St. Louis. When first called to her I found two-thirds of the womb outside of the os externum vaginæ. Thus she had been since the birth of her last child, three years ago. The bowels were costive constantly, and the irritation of the bladder was almost unbearable.

The second case is that of Mrs. G., age 31, mother of five children. Has led a life of care and trouble, compelled by poverty to labor constantly. On examination I found *more* than two-thirds of the uterus to be external to the mouth of the vagina, and the former mucous membrane covering the uterus and vagina changed to true skin. Bowels costive, and constant dribbling of urine. She had suffered in this way for nearly five years.

The last case is that of Mrs. L., age 43, a woman of rather easy virtue, to say the least. Ceased menstruating two years ago. *Has had three abortions produced on her.* On examination of this case I found the uterus just down to the opening of the vagina, while both the anterior and posterior walls of the former protruded about two inches, causing much soreness and frequent desire to urinate.

In all of these cases, as the only means of sure relief, the operation of sewing up the perineum was performed. In every case the success was perfect. In the first one, that of Mrs. B., I had some difficulty in my *after treatment* occasioned by the infiltration of urine which occurred. This was obviated, and I am pleased to report the *entire success* of these three cases, affording an example of the perfect cure of one of the most troublesome affections to which women are subject. Yours, truly,

E. R. HUTCHINS, M.D.

